# Comparative analysis of proteomes and transcriptomes revealed the molecular mechanism of development and nutrition of *Pleurotus giganteus* at different fruiting body development stages

**DOI:** 10.3389/fnut.2023.1197983

**Published:** 2023-07-21

**Authors:** Hailong Yu, Ning Jiang, Miaomiao Yan, Xuan Cheng, Lujun Zhang, Dandan Zhai, Jianyu Liu, Meiyan Zhang, Chunyan Song, Hao Yu, Qiaozhen Li

**Affiliations:** ^1^National Engineering Research Center of Edible Fungi, Institute of Edible Fungi, Shanghai Academy of Agricultural Sciences, Shanghai, China; ^2^Shandong Provincial Key Laboratory of Applied Mycology, School of Life Sciences, Qingdao Agricultural University, Qingdao, Shandong, China; ^3^Engineering Research Centre of Chinese Ministry of Education for Edible and Medicinal Fungi, Jilin Agricultural University, Changchun, Jilin, China; ^4^Agricultural Specialty Industry Development Center, Qujiang, Zhejiang, China

**Keywords:** edible mushroom, *Pleurotus giganteus*, transcriptome, development, proteome

## Abstract

*Pleurotus giganteus* is a commercially cultivated high-temperature mushroom. Investigating the molecular mechanism of fruiting body development will help us to better understand the regulation of substrates and energy in this process. However, little information has been reported on the development and nutrients of the *P. giganteus* fruiting body. In the present study, *P. giganteus* is cultivated in a climate chamber, and comparative transcriptome, proteome, and nutritional analysis of *P. giganteus* fruiting bodies were performed. Our results revealed that Cytochrome P450 monooxygenases and hydrophobin proteins play important roles during the differentiation in the elongation stage. Later, carbon metabolism dominate the fruiting body metabolism and genes related to the carbohydrate metabolic process, glycolytic process, and gluconeogenesis were up-regulated in the mature fruiting bodies. The up-regulation of carbohydrate substrates utilization CAZymes genes and inconsistent protein expression in pileus indicated a reverse transportation of mRNA from the fruiting body to vegetative mycelia. In addition, protein concentration in the pileus is higher than that in the stem, while the stem is the major nitrogen metabolic and amino acid synthetic location. The integrated transcriptomic, proteomic, and nutritional analysis indicated a two-way transportation of substrates and mRNAs in *P. giganteus*. Stem synthesizes amino acids and transported them to pileus with reducing sugars, while pileus induces the expression of substrate degradation mRNA according to the needs of growth and development and transports them in the other direction.

## Introduction

*Pleurotus giganteus* is a high-temperature wood-rotting edible mushroom. The optimal temperature for *P. giganteus* fruiting body production is around 25–32°C. Most commercially cultivated mushrooms are low-temperature or middle-temperature mushrooms, therefore, not suitable for non-industrial cultivation in summer. As a high-temperature mushroom, *P. giganteus* could cultivate in the greenhouse in summer, when most mushrooms are not suitable for cultivation. Therefore, it was widely welcomed by individual farmers. *P. giganteus* favored the consumers for its culinary tastes and health benefits (1). *Pleurotus giganteus* is rich in carbohydrates, and total protein (2), has unsaturated fatty acids (such as oleic acid, linoleic acid, and eicosadienoic acid), and lower saturated fat. Freeze-dried *P. giganteus* fruiting bodies showed hepatoprotective effects against thioacetamide-induced liver injury in rats (3). Phan et al. reported that the phenolics in *P. giganteus* showed antioxidant activity *in vitro* and the mushroom extracts presented neuritogenic activity (2). The lipid extracts from ethyl acetate fraction significantly inhibited the growth of *Candida* species, which could be further explored as antifungal agents against *Candida* species (4). In addition, *P. giganteus* also show anti-inflammatory activity, antidiabetic, antiproliferative, antidengue, and genoprotection activities (1), suggesting that *P. giganteus* has a health benefit for consumers.

The *P. giganteus* fruiting body shape of the harvest maturity is like a goblet and funnel with a long stipe and round flat pileus. The pileus has soft, meaty, and tender textures, which is suitable for cooking like other mushrooms. However, the stipe of the harvest-mature fruiting body of *P. giganteus* has an inedible hard shell, which has to be shaved off before cooking. Therefore, it is necessary to analyze genes and protein expression during the development of the *P. giganteus* the fruiting body. This information could help us better understand the molecular mechanism underlying nutrients synthesis and fruiting body texture changes (1, 5).

The growth and development of *P. giganteus* could be divided into three main phases: phase I (vegetable growth of mycelia in cultivation bag, including physiological maturation stage), phase II (transition phase or primordial inducing phase), and phase III (fruiting body development phase, from primordial to mature fruiting body). In phase I, *P. giganteus* secreted lignocellulolytic enzymes into the solid media. Polymers of solid substrates were depolymerized by these enzymes into small molecules, which were absorbed by *P. giganteus* mycelia. After the mycelium occupies the whole cultivation bag, *P. giganteus* undergo a further cultivation period to reach physiological maturation (6). In phase II, the cultivation environment was dramatically changed. The soil was added to the top of the open cultivation bag (7), the water that was lost during phase I was replenished, temperature stimulation was provided, and light exposure was necessary. Changed environmental factors stimulate the formation of hyphal knots and primordia. In phase III, primordial differentiated into pileus and stipe, which finally form a mature fruiting body. In the present study, phase III of *P. giganteus* fruiting body development was selected for further analysis.

The fruiting body development of many edible mushrooms has been investigated by transcriptomic or proteomic analysis, including *Dictyophora indusiata* (8, 9), *Agrocybe aegerita* (10), *Lentinula edodes* (11, 12), *Hypsizygus marmoreus* (13, 14), *Flammulina filiformis* ((15, 16)), *Stropharia rugosoannulata* (17, 18), *Pleurotus tuoliensis* (19), *Pleurotus eryngii* (20, 21), *Botrytis cinerea* (22), *Ganoderma lucidum* (23), et al. In a previous study, we found that the protein expression patterns between the cap and stipe of mature *Stropharia rugosoannulata* show a lower correlation (with Pearson correlation coefficients between 0.50 and 0.58) with each other. Proteins related to carbon metabolism, energy production, and stress-response-related biological processes had higher abundances in stipe tissue compared with those in the cap, while proteins associated with fatty acid synthesis and mRNA splicing showed higher expression levels in the cap (18). Our previous research about *H. marmoreus* indicated that proteins involved in biomass increase, cell proliferation, signal response, and differentiation might play key roles in fruiting body development (13). Omics research of the fruiting development could help us understand the molecular mechanism underlying this important biological process and mine key genes responsible for development. However, the differential expression analysis of *P. giganteus* development was not reported to date.

Recently, a high-quality genome of *P. giganteus* was sequenced and assembled by our lab (7). The genome size of *P. giganteus* is ~ 40.00 Mb in 27 contigs. Comparative genomic and phylogenetic analysis revealed that *P. giganteus* belongs to *Pleurotus* species with a closer relationship to *Pleurotus tuber-regium* and *Pleurotus citrinopileatus*, which also have a funnel shape fruiting body. CAZymes analysis confirmed the robustness of the lignocellulose degradation capacity of *P. giganteus*. However, the development information about this mushroom especially at a molecular level is still scanty. In the present study, comparative transcriptome and proteome analysis of the *P. giganteus* fruiting body in four different developing stages were performed to obtain a comprehensive and systematic understanding of the morphological development process and nutritional changes of the fruiting body underlying molecular mechanisms.

## Results

### Six groups of *Pleurotus giganteus* fruiting bodies among four stages during fruiting body development

**Figure 1:**
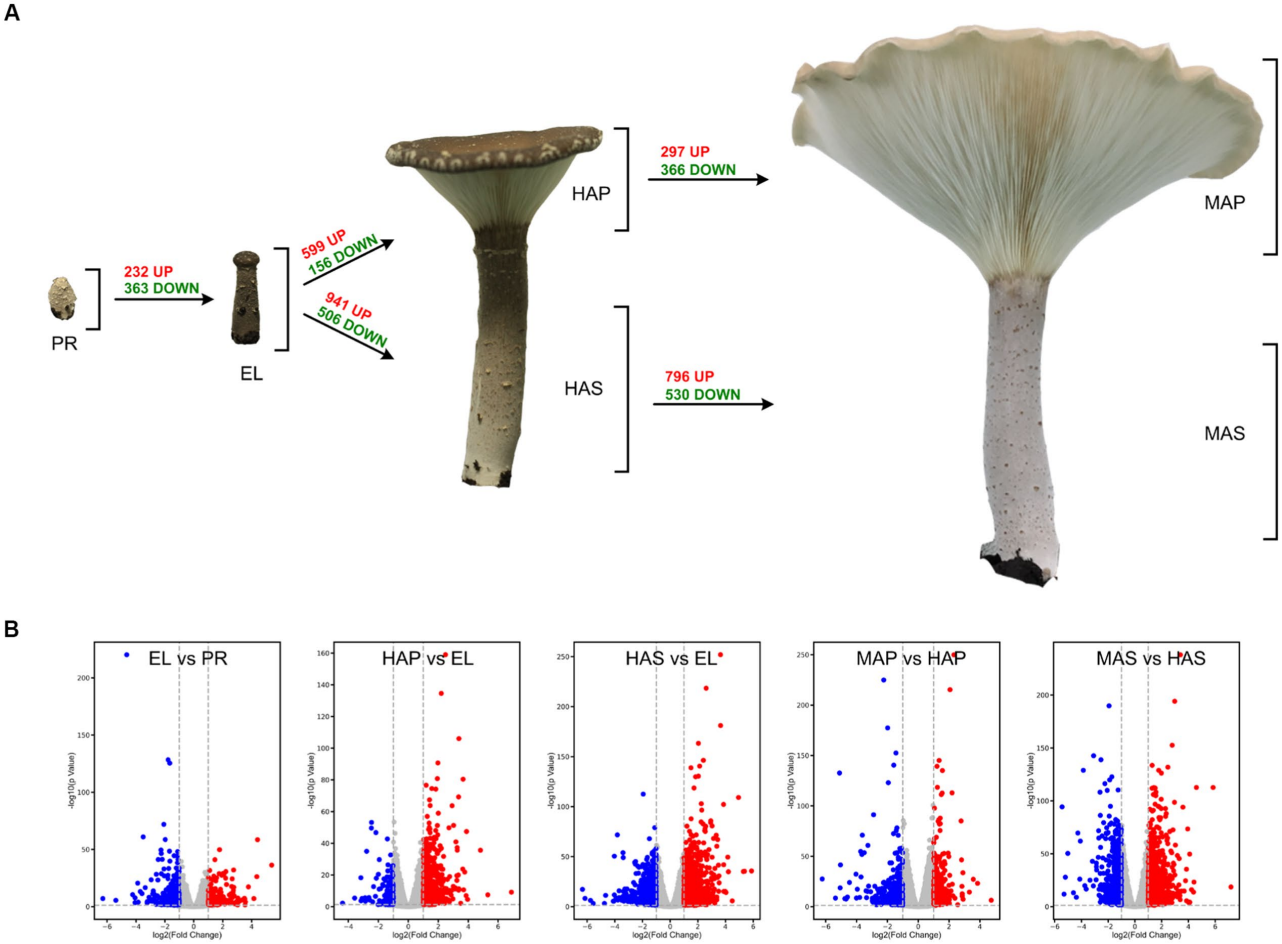
The different developmental stages of *Pleurotus giganteus*. Volcano plots indicated that differential gene expressions were detected in transcriptome analysis. **(A)** *P. giganteus* fruiting bodies at different development stages. **(B)** Volcano plots representing differential expressed genes between different groups.

The reproductive developmental processes of *P. giganteus* can be divided into four stages: primordium (PR) stage, elongation (EL) stage, harvest (HA) stage, and maturation (MA) stage. Production of *P. giganteus* strain Shenxun1 was carried out in the polyethylene bag. One thousand polyethylene bags were cultivated in an artificial climatic chamber at 22–23°C for ~60 days. After the maturation of the mycelia, the temperature was increased to 27°C (day 0) for primordia induction and fruiting body production. The mycelia kinked and differentiated into primordium in 7 ~ 10 days after soil covering (Figure 1). Then, the stipe of the fruiting body elongated while the pileus did not spread (EL stage). About 14–16 days after induction, the fruiting body developed into a funnel-shaped pileus, and the edge of the cap inward roll. Fruiting bodies were harvested at this stage. The fruiting body is fully mature at 19–21 days. At this stage, the pileus shape is like a large funnel and the edge of the cap is flat or rolled up (Figure 1). To better understand the development and nutrient synthesis processes of *P. giganteus*, samples (three biological replicates) from the four reproductive developmental stages were collected and used for transcriptome and proteome analysis.

### Overview of the transcriptome sequence data

**Figure 2:**
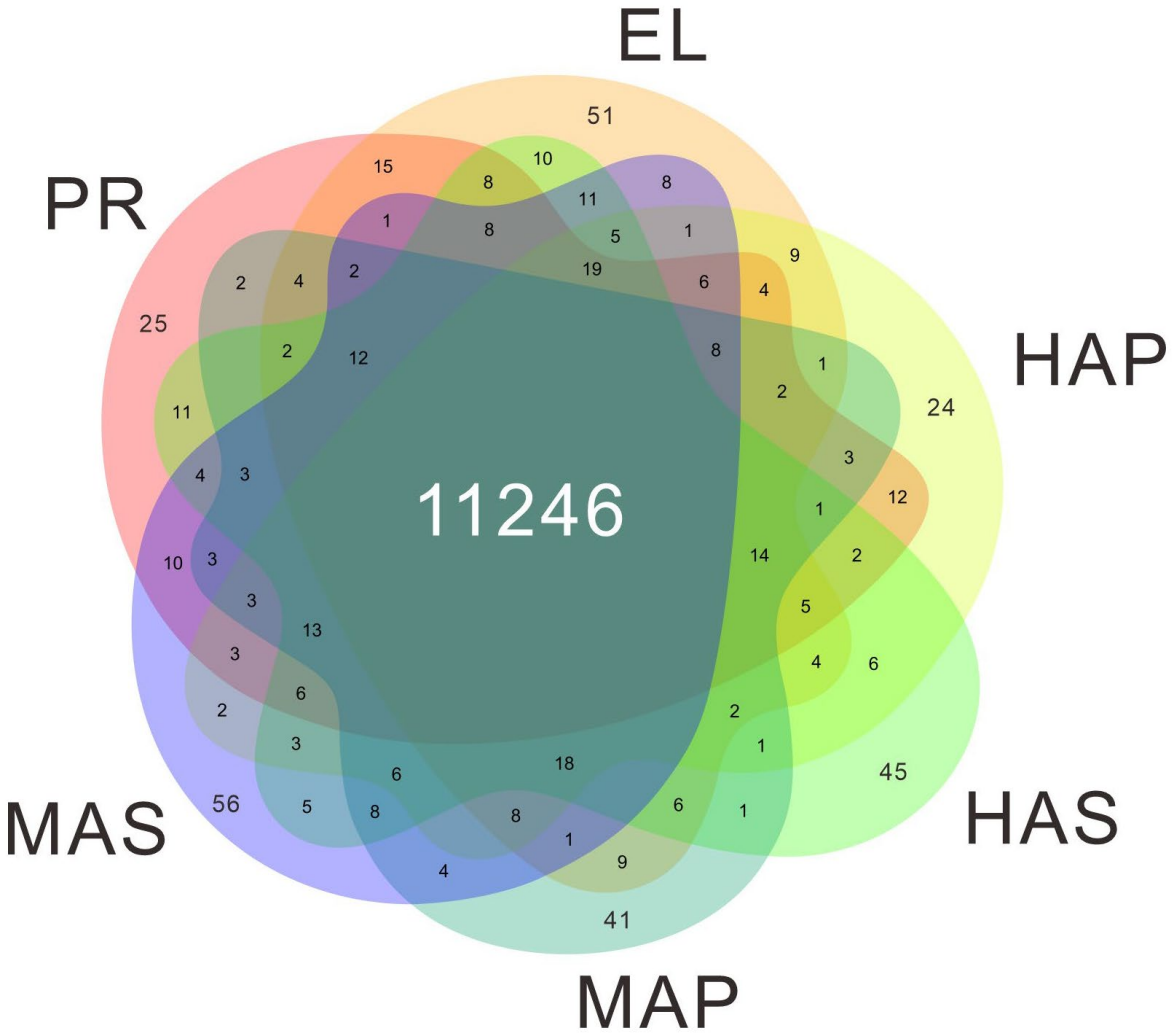
Venn diagram showing the gene identified in the transcriptome data.

The genome of *P. giganteus* has been sequenced by our team recently (7), and we here report an updated version (Pgig-A-v02). The improvements in the updated assembly are reported in the methods part and the new version of genome annotation could be obtained from the Laboratory of Mushroom Precision Breeding (https://file.mushroomlab.cn/). RNA-Seq analysis was performed using the Illumina platform. After data filtering and trimming, 716,182,962 clean reads with a total of 107 G clean data were obtained from the 18 RNA-Seq samples (3 biological replicates for each group). The filtered RNA-Seq reads were mapped into *P. giganteus* genome using Hisat2 software and the reads number mapped to each gene was calculated using Rsubread package. The average mapping rate is 74.78%. A total of 11,822 genes were detected in the 18 RNA-Seq samples, among which 11,246 genes were identified in all 6 samples (Figure 2; Supplementary Table S1).

**Figure 3:**
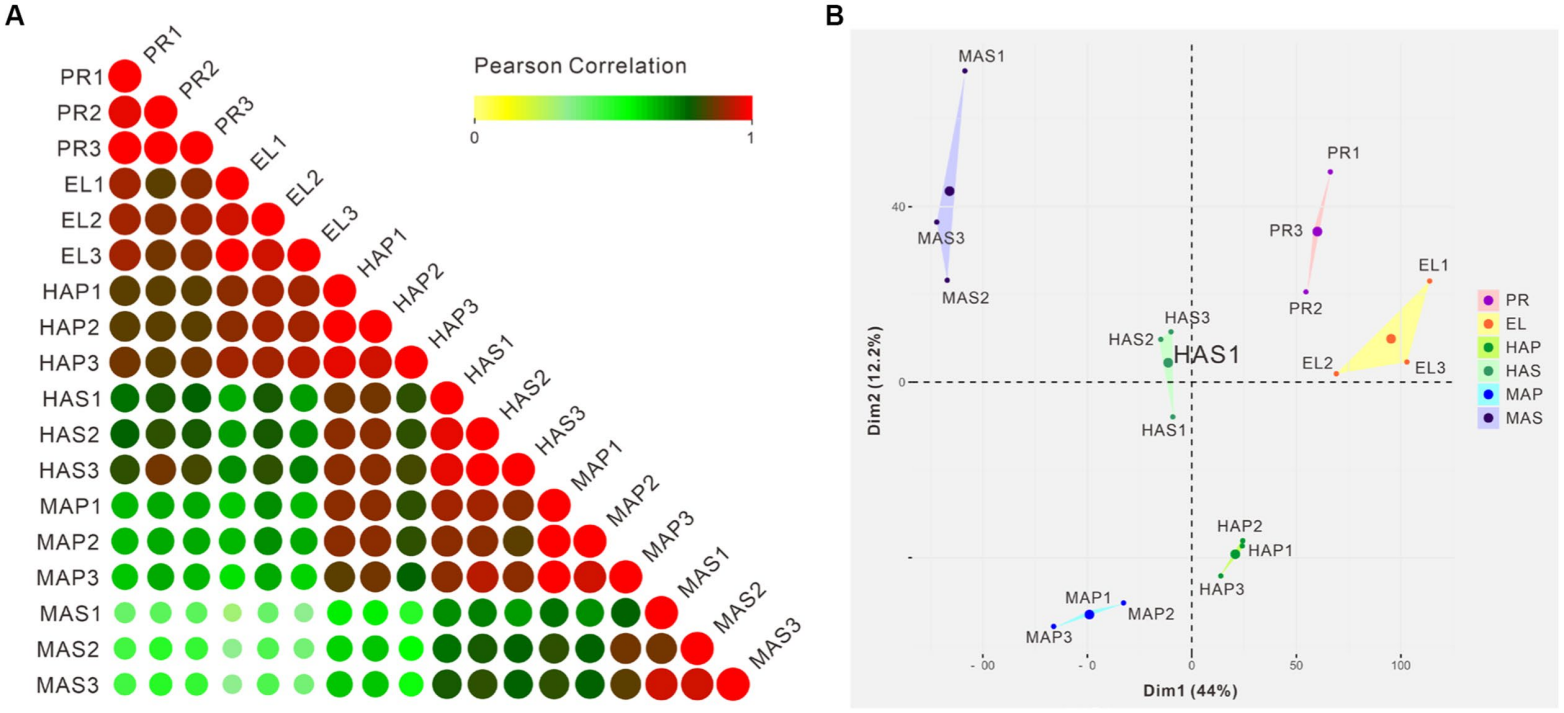
Correlation analysis and PCA of 18 samples from transcriptome samples. **(A)** Pearson correlation analysis of 18 samples from 6 groups based on transcriptomic data. **(B)** PCA scores of the first two principal components based on individual transcriptomic data from 6 groups.

Pearson correlation analysis showed that high correlation coefficients were distributed near the diagonal and lower correlation coefficients were distributed at the left bottom (Figure 3A). PCA showed similar results that the PR group and the EL group were mainly distributed in the right upper quadrant, samples from the HA stage were distributed in the middle, and samples from the MA stage were distributed in the left (Figure 3B). Besides, a spatial-dependent distribution could be observed in the PCA results. Samples from the pileus were distributed in the lower quadrant, while samples from the stem were distributed mainly in the left upper quadrant (Figure 3B). The results indicated that adjacent developmental stages showed similar gene expression patterns and the gene expression patterns kept changing during the fruiting body development in *P. giganteus*.

### Temporal gene expression across *Pleurotus giganteus* development

One-way ANOVA analysis based on TMM revealed that the expression of 8,847 genes (74.8% of identified genes) significantly changed (FDR < 0.05) during fruiting body development (Supplementary Table S1), which indicated that most of the genes are involved in the differentiation in the development of *P. giganteus* fruiting body. The differential expression of genes during different developmental stages was analyzed using edgeR software (Figure 2A; Supplementary Table S2–S8). The results indicated that the highest number of differentially expressed genes (DEGs) in the transition from EL group to HAS group (941 up-regulated, 506 down-regulated), followed by HAS group to MAS group (796 up-regulated, 530 down-regulated). The lowest number of DEGs in the transition from the PR group to the EL group (232 up-regulated, 363 down-regulated), followed by the HAP group to the MAP group (297 up-regulated, 366 down-regulated; Figures 2A,B).

**Figure 4:**
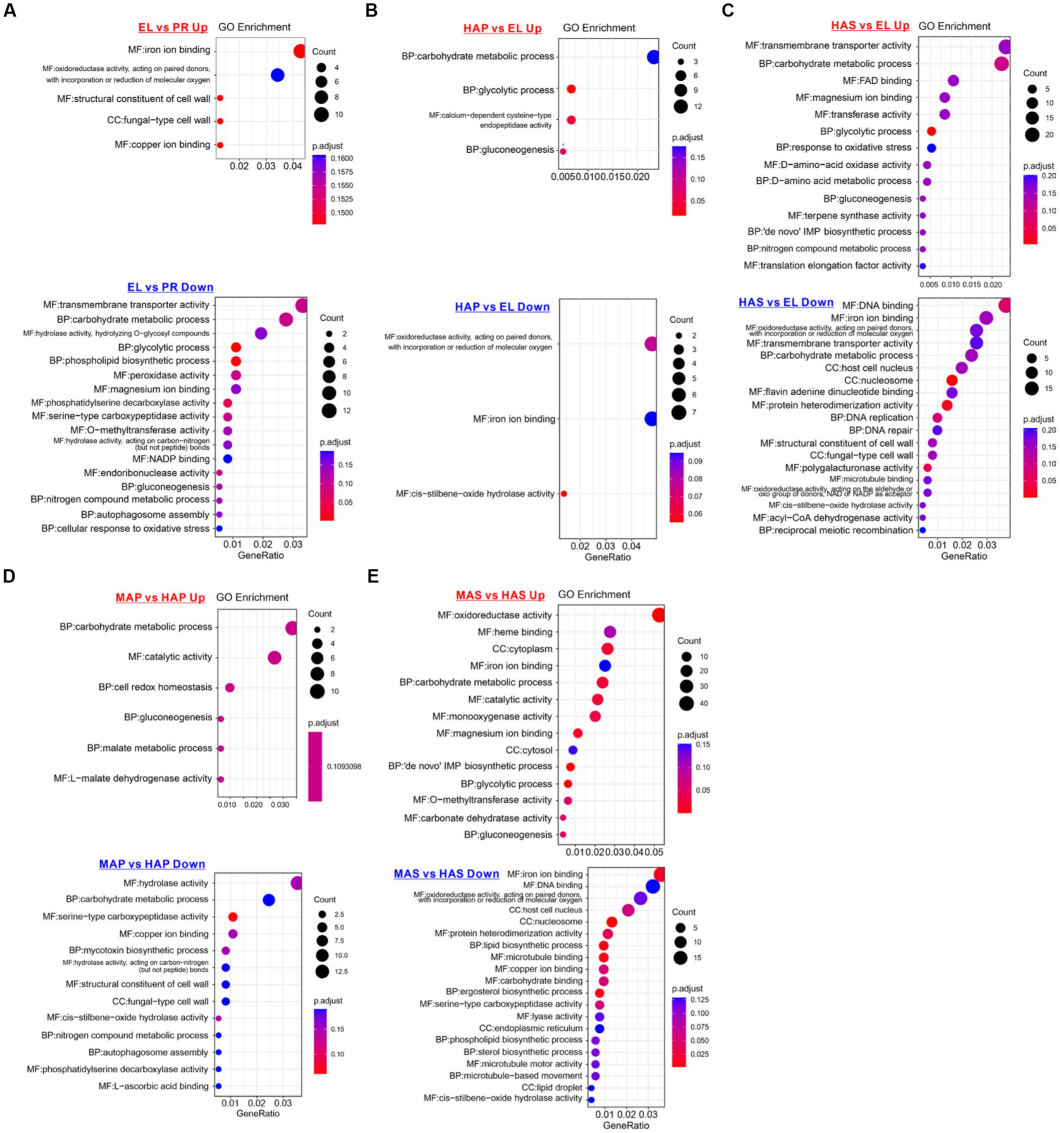
Enriched gene ontology (GO) terms of up-regulated and down-regulated DEGs between different developmental stages. **(A)** Enriched GO terms of DEPs between the EL group and PR group. **(B)** Enriched GO terms of DEPs between the HAP group and EL group. **(C)** Enriched GO terms of DEPs between the HAS group and EL group. **(D)** Enriched GO terms of DEPs between the MAP group and HAP group. **(E)** Enriched GO terms of DEPs between MAS group and HAS group.

To investigate the molecular basis of fruiting body development, gene ontology (GO) enrichment analysis of the DEGs was performed. As we can see from Figure 4A, GO terms of iron binding (mainly Cytochrome P450 monooxygenase) and structural constituent of the cell wall (hydrophobin proteins) were enriched in the up-regulated DEGs in the EL group. GO terms of transmembrane transporter activity, carbohydrate metabolic process, glycolytic process, peroxidase activity, and magnesium ion binding were enriched in the down-regulated DEGs in the EL group compared with the PR group (Figure 4A).

On the contrary, compared with the EL group, carbohydrate metabolic process, glycolytic process, and gluconeogenesis were enriched in the up-regulated DEGs in both HAP and HAS groups. In addition, GO terms of magnesium ion binding, response to oxidative stress, D-amino-acid metabolic, nitrogen compound metabolic process, and translation elongation factor activity were enriched in up-regulated DEGs in HAS groups. The results indicated that carbon metabolism is more activated in the fruiting body in the harvest stage than in the elongation stage, while nitrogen metabolism is more active in the stem (Figures 4B,C).

### Nutrients of *Pleurotus giganteus* fruiting body

As described above, the texture of the pileus and the stem of the *P. giganteus* fruiting body are quite different. The stem of the harvest-mature fruiting body has an inedible hard shell. Therefore, only pileus was collected for sale. To evaluate the nutritional values of pileus and stem, the protein, lipid, fiber, and sugar were analyzed. All four nutrients are significantly different between pileus and stem. Pileus tissue has higher protein and lipid concentrations than the stem (Table 1). However, the stem has higher crude fiber and reducing sugar content. Higher fiber concentration is consistent with the hard shell of the stem tissue.

**Table 1:** Comparison of nutritive components between pileus and stem of *Pleurotus giganteus* fruiting body at harvest stage.

	Total protein (g/100 g dry matter)	Lipid (g/100 g dry matter)	Crude fiber (g/100 g dry matter)	Reducing sugar (g/100 g dry matter)
Pileus	27.9 ± 0.36	1.1 ± 0.01	7.4 ± 0.1	7.09 ± 0.065
Stem	17.3 ± 0.1	0.4 ± 0.01	10.83 ± 0.115	9.23 ± 0.119

### Differential expression of genes and proteins of fruiting body in harvest stage

**Figure 5:**
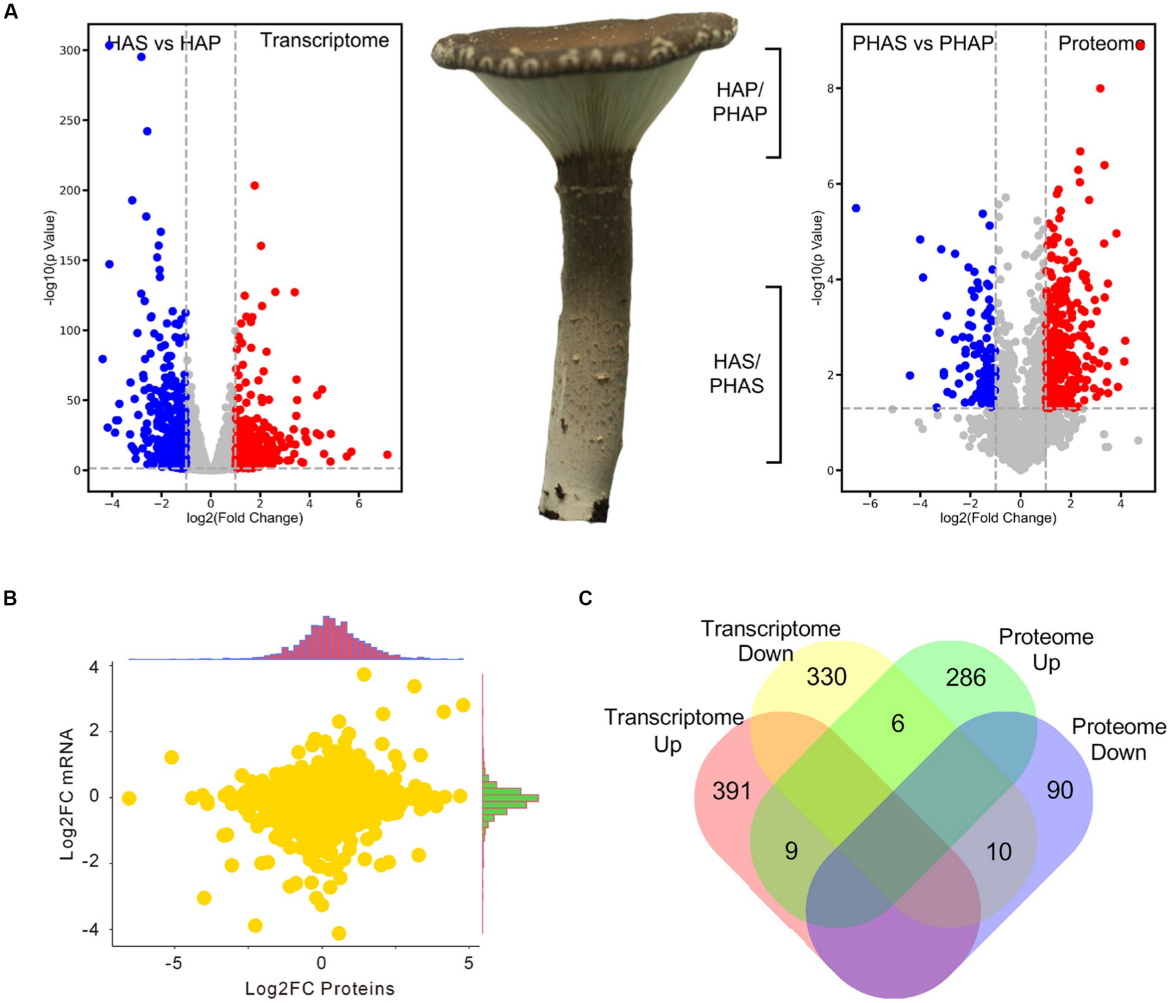
Volcano plot showing the differential expression of genes and proteins in stipe and pileus of *P. giganteus* fruiting body in HA stage. **(A)** Volcano plot representing the differentially expressed genes or proteins between pileus and stem samples. **(B)** Correlation analysis between transcriptome and proteome data. **(C)** Venn diagram analysis of the up-/down-regulated genes(proteins) in transcriptome and proteome data.

Compared with stem (HAS group), 400 genes were upregulated and 346 genes were significantly down-regulated in the pileus of the fruiting body (HAP group). In proteomic analysis, compared with the PHAS group, 301 proteins were significantly up-regulated and 100 proteins were down-regulated in the PHAP group (Figure 5A; Supplementary Table S10). The correlation between the fold change of HAP vs. HAS groups in transcriptome and the fold change of PHAP vs. PHAS groups in proteome was analyzed (Figure 5B). Samples of HAP/HAS and PHAP/PHAS groups were from the same growth stage; however, the correlation (0.15) between transcriptome and proteome data was low (Figure 5B). Among the differentially expressed genes and proteins, only 9 genes were both up-regulated and 10 were both down-regulated in transcriptomic and proteomic analysis. Six genes were up-regulated in the PHAP group but down-regulated in the HAP group. Other 1,097 (97.8%) DEPs or DEGs were only identified in proteomic analysis or transcriptomic analysis (Figure 5C). The results indicated that no correlation was observed between the transcriptome and proteome data.

**Figure 6:**
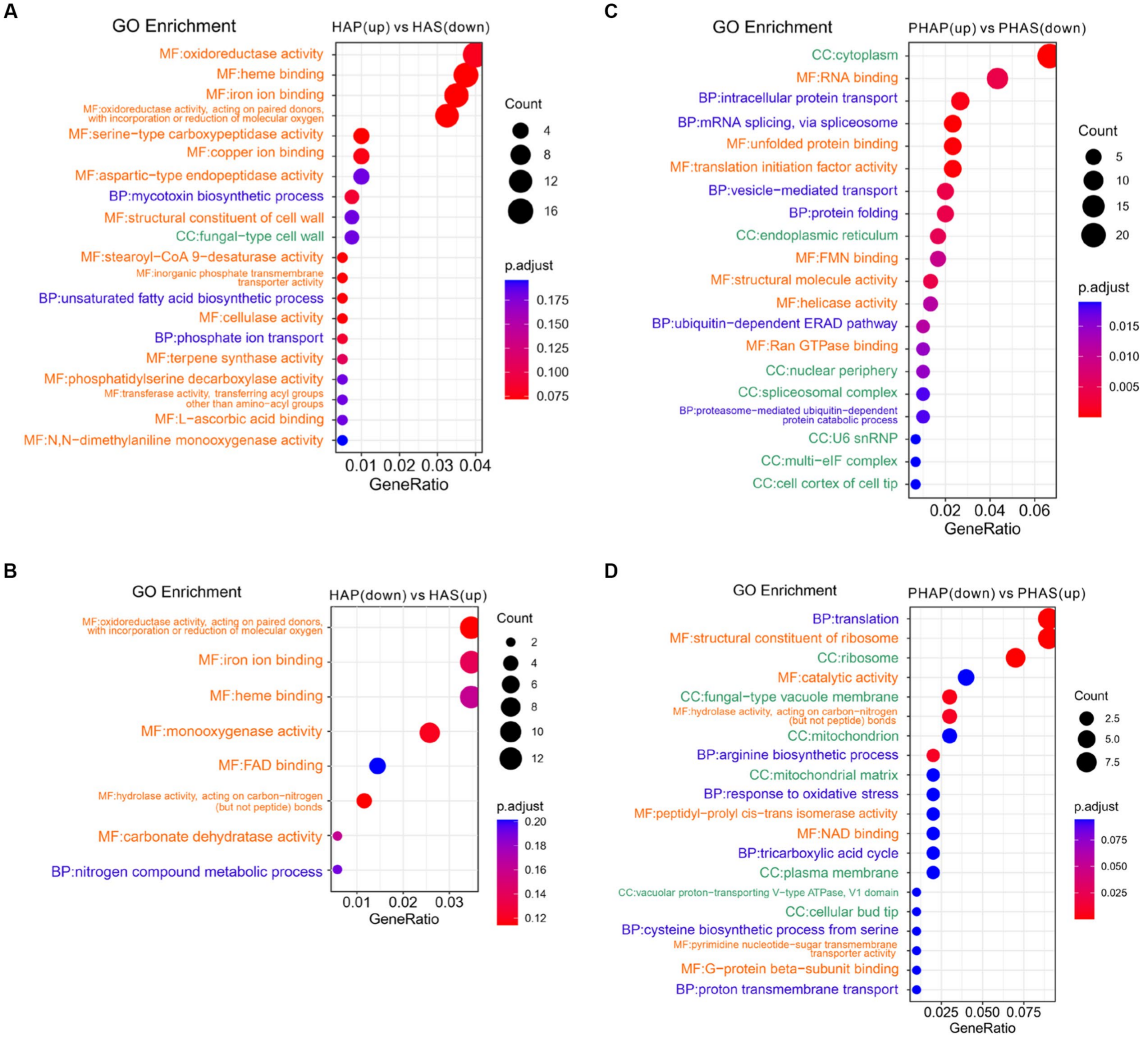
Enriched GO terms of up-regulated and down-regulated DEGs and DEPs between pileus and stem tissue in the harvest stage. **(A)** Enriched GO terms of up-regulated DEGs in the HAP group. **(B)** Enriched GO terms of down-regulated DEGs in the HAP group. **(C)** Enriched GO terms of up-regulated DEPs in the PHAP group. **(D)** Enriched GO terms of down-regulated DEPs in the PHAP group.

Differentially expressed genes and DEPs identified between pileus and stem in the harvest stage were analyzed using clusterProfiler. GO enrichment analysis revealed that GO terms of oxidoreductase activity, heme binding, ion binding, and cellulase activity were enriched in up-regulated DEGs in HAP compared with HAS group (Figure 6A). Interestingly, GO terms of oxidoreductase, ion binding, heme binding, hydrolase activity, and carbonate dehydratase were enriched in down-regulated DEGs in the HAP group compared with HAS group (Figure 6B). GO terms of cytoplasm, RNA binding, intracellular protein transport, mRNA splicing, splicesome, and unfolded protein binding was enriched in up-regulated DEPs in the PHAP group compared with the PHAS group. GO terms of translation, ribosome, catalytic activity, fungal-type vacuole membrane, and hydrolase activity were enriched in down-regulated DEPs in the PHAP group compared with the PHAS group (Figures 6C,D). To further investigate the differences between pileus and stem, KEGG enrichment analysis was performed to identify the different pathways enriched in the different parts of the fruiting body (Supplementary Figure S1). The results of the KEGG enrichment are in line with the above results and our previous study in *Stropharia rugosoannulata* (18). Protein processing in endoplasmic reticulum pathway was enriched in up-regulated DEPs in the PHAP group (Supplementary Figure S2). The results confirmed that pileus is the main protein synthesis area in line with the high protein concentration in pileus. In addition, the oxidative phosphorylation pathway was enriched in up-regulated DEPs in the PHAP group (Supplementary Figure S3). The carbon metabolism and energy production pathways were also enriched in the up-regulated proteins in *S. rugosoannulata* in our previous study (18), which further confirmed that the carbon metabolism process is dominant in the mature fruiting body.

## Discussions

Mushroom fruiting bodies are highly complex multicellular structures in fungi, which protect, produce, and disperse sexual spores (24, 25). Fruiting bodies are the main edible parts of mushrooms. For most mushrooms, the ripening time from primordial to harvested (commercial-mature) fruiting body is only 7–10 days, which is only a small part of the overall production cycle. However, this period has a great influence on the commercial quality of the mushrooms. Therefore, mushroom fruiting body development is one of the core research field of mushrooms. The development of mushroom fruiting bodies has extensively been studied at the omics and physiological level (26–38). Our current knowledge of the biological process and molecular mechanism of fruiting body development in *P. giganteus* is limited. In this study, comparative transcriptomic and proteomic analyses of *P. giganteus* fruiting body development were performed for the first time.

If we assume that the number of DEGs between two groups represents the differentiated degree between the two samples, the results indicated that the degree of differentiation between the primordium stage and the elongation stage was low while the degrees of fruiting body differentiation between elongation stage, harvest stage, and maturity stage was high, especially in the stem tissue. The morphological change is inconsistent with the number of DEGs. The pileus and stem tissues differentiated from the elongation stage and the gills were first developed in the harvest stage. Continuous development of the fruiting body could be observed from the harvest stage to the maturity stage. The color and shape of the fruiting body were changed and harder shells and flakes were observed in the stem at this stage. The differential expression of genes could reflect these traits. Liu et al. reported that transcriptomic analysis of *Flammulina filiformis* showed that more DEGs were observed between pileus tissue than stem tissue (15). Different from *P. giganteus*, the stem of *F. filiformis* is edible and does not have a dark color and hard shell. The results indicated the metabolic processes in the *P. giganteus* stem are more complex than those in *F. filiformis*.

Compared with the PR group, genes encoding Cytochrome P450 monooxygenase and hydrophobin proteins were significantly enriched in up-regulated genes in the EL group. The expression of Cytochrome P450 and hydrophobin proteins during fruiting body development is conserved in fungal (15, 25). Liu et al. (15) reported that genes encoding hydrophobins were significantly up-regulated in the primordium stage compared with vegetative mycelium. Hydrophobins were self-assembled into a rodlet layer on the cell surface, conferring hydrophobic surfaces to hyphae that hinder soaking of fruiting bodies with water. Hsu et al. (39) reported Cytochrome P450 genes were strongly expressed during the formation of fruiting bodies. The mutation of a cytochrome P450 gene in *Coprinus cinereus* affected the pattern formation in the development of fruit body primordia (40). The expression of several cytochrome P450 genes was significantly changed during the development of the *Hypsizygus marmoreus* fruiting body (14). All these results indicated that these Cytochrome P450 and hydrophobin genes play key roles in the initial developmental stage in *P. giganteus* fruiting body development.

Carbon metabolism is the central metabolism during fruiting body development, especially in the pileus. The volume of pileus changed the most during the development. As we know that the main element in fungal mycelia is carbon. Indeed, the expression of carbon metabolism-related genes becomes more active with the development of the *P. giganteus* fruiting body (Figure 4). The expression of carbon metabolism genes is related to the increasing volume of the fruiting body.

It is worth noting that several inconsistent data were observed in the present study, including the low correlation between gene expression in the transcriptional level and translational level and the negative correlation between protein concentration and nitrogen metabolic. Poor correlation between transcriptomic and proteomic data revealed that the gene expression at transcriptional and translational levels is quite different. This kind of poor correlation was common and also observed in other research (41, 42). The concentration of proteins was influenced by many factors, including mRNA expression level, post-transcriptional regulation, translation regulation, and post-translation modification. In addition, mRNA and protein transportation between different cells will also influence the expression levels of the same gene. Different sample processing procedures, especially for proteomic analysis, could also influence the correlation between transcriptome and proteome data. However, the correlations between different protein extraction and peptide digestion methods are more than 0.9 (43). Therefore, some reasons beyond sample processing must have played a key role in the poor correlation.

Unexpectedly, from the analysis of gene and protein expressions between pileus and stem in the harvest stage (Figure 5), we found that 70 (17.5% of all 400) up-regulated DEGs in the HAP group were secreted proteins, while only 13 (4.3% of all 300) up-regulated DEPs in PHAP group were secreted proteins. This contradiction between high proportion of secreted proteins in DEGs and low proportion of secreted proteins in DEPs could be explained by transportation of mRNA.

**Figure 7:**
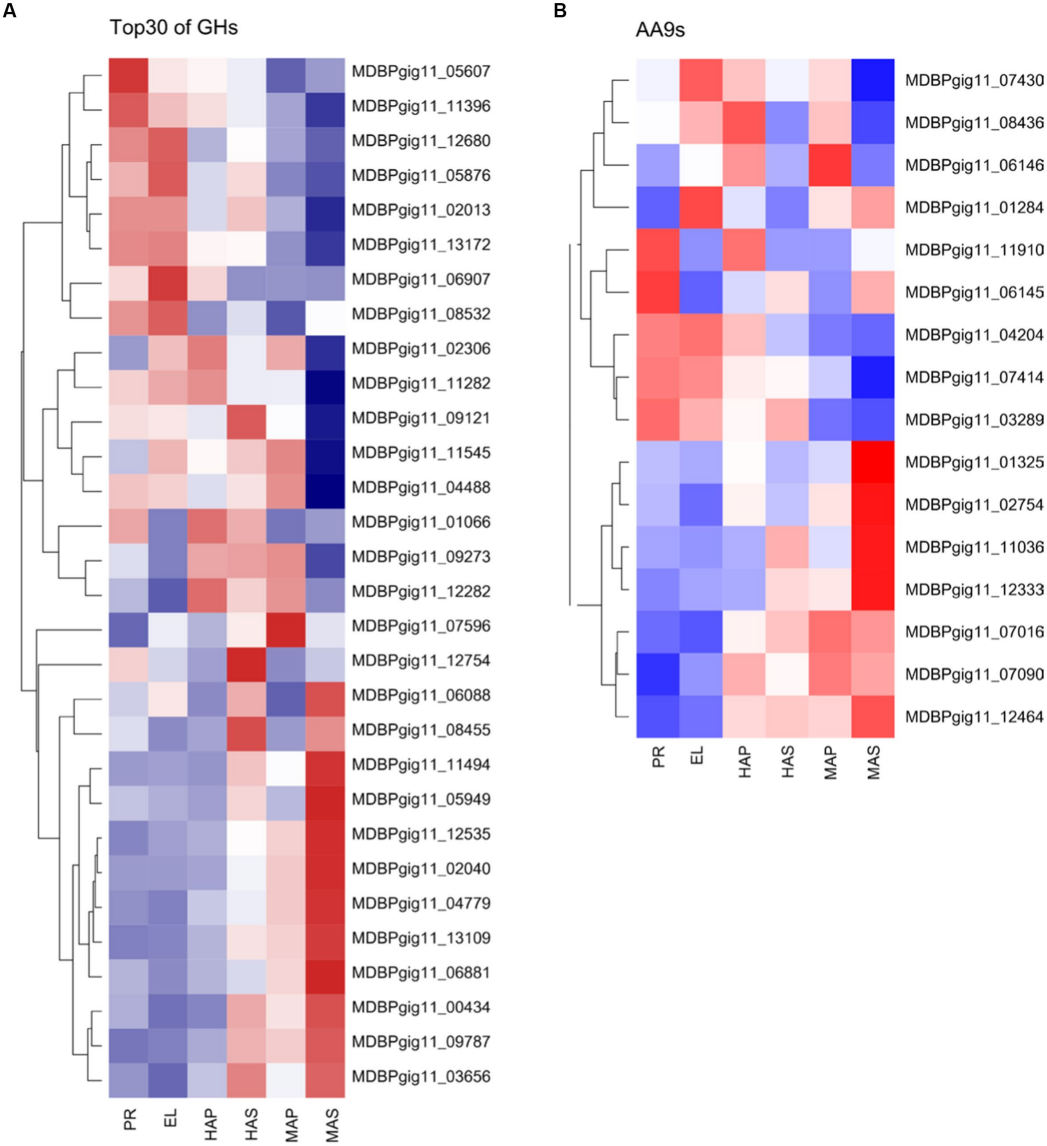
Differentially expressed CAZymes in transcriptome analysis. **(A)** Top 30 of the highly expressed differentially expressed genes of GHs family. **(B)** Differentially expressed genes of AA9s family.

CAZymes play key roles in substrate utilization in edible mushrooms which provided carbon and nitrogen sources for mycelia growth and fruiting body development. Besides, CAZymes also play important roles in fruiting body development as reported in omics studies (11, 44, 45). We analyzed all the 489 predicted CAZymes in the *P. giganteus* genome, 176 (36.0%) CAZymes genes were among the DEGs described in Figures 1 and 5 (Supplementary Table S10). CAZymes analysis between different groups indicated that most lignin modification enzymes (LMEs) in AA1 and AA2 families down-regulated with the development of the fruiting body. However, most of the CAZymes related to cellulose and hemicellulose degradation (from AA9, GH11, GH5, GHJ7, GH9, GH51, GH22) were up-regulated with the development of the fruiting body. As shown in Figure 7A, cellulose and hemicellulose degradation genes from GHs family were mainly upregulated in PR stage and maturation stage (MDBPgig11_11494, MDBPgig11_05949, MDBPgig11_02040, MDBPgig11_13109, MDBPgig11_04488, MDBPgig11_04779). Lytic polysaccharide monooxygenases (LPMOs) from AA9 families are responsible for the degradation of cellulose, most of the LPMOs were also highly expressed in PR and maturation stages (Figure 7B), which is in consistent with that of DEGs from GHs families.

**Figure 8:**
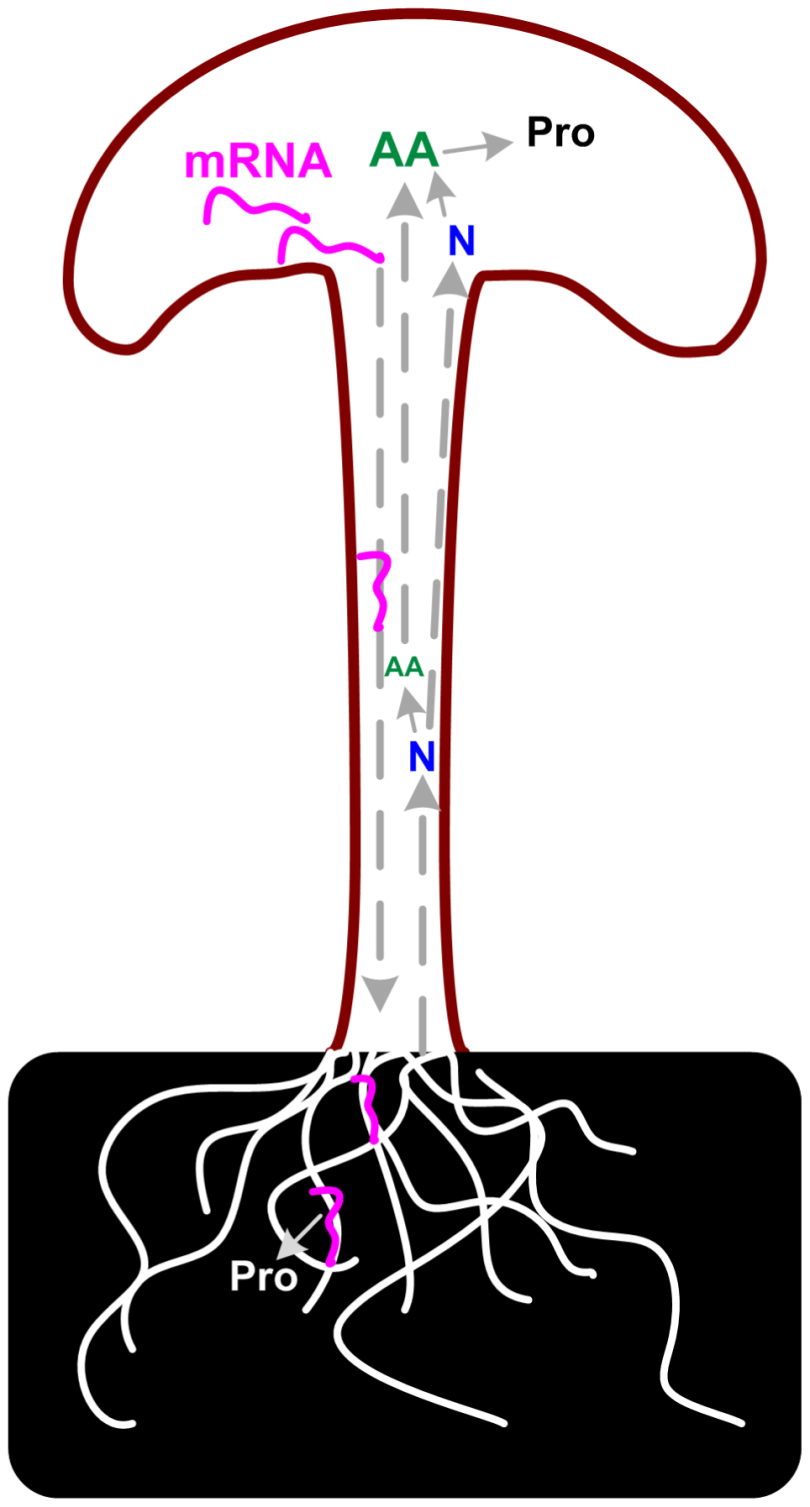
Scheme of proposed carbon and nitrogen metabolism in *P. giganteus* fruiting body.

Considering the poor correlation between transcriptome and proteome data, the unusually high proportion of secreted DEGs in fruiting body samples, and the CAZymes analysis results, we assumed that there is a long-distance reverse transportation mechanism of these mRNAs from the fruiting body to substrate mycelia as in plant (46). Up-regulation of (hemi)cellulose CAZymes and down-regulation of lignin modification enzymes during fruiting body development has also been reported in *Agaricus bisporus* and *Ganoderma lucidum*, which revealed a two-step lignocellulose degradation strategy in these mushrooms (47, 48). Both studies detect the transcriptome of mycelia in solid media and the gene expression of these genes in mycelia is consistent with the abundance of the secreted proteins. However, none of these studies explained the regulation mechanism of the expression of these CAZymes during development. Similar results were also reported in other mushrooms, that the expression of carbohydrate hydrolases was highly expressed in the fruiting body of several mushrooms (25). As described by Krizsán et al. that the targets of these CAZymes families in fruiting bodies are currently unknown. Considering the correlation between the secreted proteins and the gene expression in the fruiting bodies, the results lead us to the hypothesis that the need for nutrients in the fruiting body could induce the expression of corresponding genes. mRNA of lignocellulose degradation genes could transport from the fruiting body to substrate mycelia and induce the secreted protein synthesis and secretion (Figure 8).

The negative correlation between protein concentration and nitrogen/amino acid metabolism also supports the above speculation to a certain extent. As we observed in proteomic analysis, DEPs related to the arginine biosynthetic process, peptidyl-prolyl cis-trans isomerase activity, and cysteine biosynthetic process from serine GO terms were up-regulated in the stem. In addition, the DEGs related to the nitrogen compound metabolic process GO term were significantly enriched in the HAP group. Compared with the EL group, nitrogen compound metabolic processes were enriched in up-regulated DEGs in the HAS group but not in the HAP group. All the results indicated that the stem is the center for nitrogen metabolism and amino acid synthesis. The synthesized amino acids might be transported to pileus to synthesize protein or transported to substrate mycelia with mRNA to synthesize secreted proteins.

There must be other factors besides transportation that influenced the expression patterns of genes and proteins in the fruiting body. The expression of the *Roc1* regulator gene, which regulates the expression of cellulose degradation genes in *Schizophyllum commune*, homologous in *P. giganteus* was not induced at any developmental stages (49). Therefore, the expression of these CAZymes might be induced by other factors, which need further investigation.

## Conclusion

Integrated transcriptomic and proteomic analysis revealed that Cytochrome P450 and hydrophobin genes play key roles in the initial developmental stage in *P. giganteus* fruiting body development. While in the mature fruiting bodies carbon metabolism is the central metabolism during fruiting body development, especially in the pileus. To obtain the source of carbon, the substrate utilization genes may be inducibly expressed and the mRNA is transported to vegetative mycelia to synthesize secreted proteins. The nitrogen metabolism and amino acid synthesis process were more active in the stem and the products might transport to pileus together with the reducing sugar from the substrates. As a result, pileus has higher protein and reducing sugar concentrations. To further investigate the observations, there is a need to better understand the regulation mechanism of these differentially expressed genes and proteins.

## Materials and methods

### Mushroom materials

Fresh primordia and fruiting bodies of *P. giganteus* strain Shenxun1 were collected from an automated cultivation plant at the Shanghai Academy of Agricultural Sciences. Samples without physical damage and uniform in size at different development stage (Figure 1A) were selected for transcriptome and proteome analysis. The fruiting body was longitudinally cut along the central axis to 2–4 mm thick sliced tissues. Tissues from three fruiting bodies were mixed and ground using liquid nitrogen. Three biological replicates were taken for one development stage. The cut samples were frozen in liquid nitrogen and stored at − 80°C for further analysis.

### Determination of nutrients

Protein and total lipid contents were determined using the Kjeldahl nitrogen method and the Soxhlet method, respectively. Polysaccharides were converted to reducing sugars by acid hydrolysis, and then the total reducing sugar was measured using the dinitrosalicylic acid (DNS) method. The crude fiber was estimated using the traditional Van Soest method (50). The ash content was weighed after the sample was heated in a muffle furnace at a temperature of 550 ± 10°C for 1 h. Amino acids were determined using Automatic Amino Acid Analyser (Hitachi, LA8080). All measurements were performed with three replicates. The data are presented as the mean values with standard deviations (mean ± SD).

### Protein extraction and digestion

Total proteins were extracted from the frozen fruiting bodies of *P. giganteus* according to the following procedures: 200 mg sample taken from the fruiting body was cut into ~ 2 mm squares and transferred into a 2 mL centrifugation tube with two 5 mm 316 stainless steel beads. After cooling with liquid nitrogen, the sample was broken using Tissue Lyser JXFSTPRP-24 L (Jingxin, Shanghai, China) at 60 Hz for 120 s. Five-hundred microliter pre-heated buffer (4%SDS, 0.1 M Tris/HCI, pH7.6) was added into the tube, and cell extracts were treated at 95°C for 10 min. Then the sample was treated by ultrasonication for 2 min (51). Cell debris was removed by centrifugation at 15000 *g* for 20 min at 4°C, and the supernatant was transferred into a new 1.5 mL tube. Protein concentration was determined using the Modified Bradford Protein Assay Kit (Sangon Biotech, Shanghai, China).

Protein digestion was performed using STrap methods as described by Zougman et al. (52). STrap-tip was prepared according to the methods described by Zougman et al. using quartz fiber filter (MK 360, Munktell) and Empore disk-C18 (Sigma) membranes. One hundred microgram proteins from each sample were reduced using DTT and alkylated using iodoacetamide. The strapping proteins were digested using 2 μg trypsin (ThermoFisher, United States) at 47°C for 60 min. Eluted desalted peptides were evaporated on an RVC 2-25 CD plus vacuum concentrator (Christ, Osterode am Harz, Germany), and stored at − 80°C for further analysis.

### LC–MS analysis and data processing

Desalted peptides were redissolved in 0.1% formic acid, and the peptide concentration was measured using a Nanodrop spectrophotometer (ThermoFisher, United States). The final peptide concentration was adjusted to 0.6 ~ 0.8 μg/μL. Label-free proteomic analysis was performed using a capillary LC-ESI-MS/MS system coupled to Orbitrap Fusion Lumos (Thermo Fisher, CA, United States) as previously described (53). The digested peptides were loaded onto the Acclaim PepMap 100 analytical column (75 μm × 15 cm, nanoViper C18, 3 μm, 100 Å) in buffer A (0.1% formic acid) and separated with a linear gradient of buffer B (84% acetonitrile, 1% formic acid) at a flow rate of 300 nL/min at 25°C: held in 5–10% buffer B for 3 min, 10–40% buffer B for 68 min, 40–100% buffer B for 4 min, and 100% buffer B for 15 min. The MS instrument was operated in data-dependent acquisition mode (DDA). For each group, three biological repeats were performed.

Protein identification from acquired MS/MS spectra raw data was performed using MaxQuant 2.0.2 (54) against version 2 of *P. giganteus* proteome database^1^ with default parameters: iBAQ label free quantification, carbamidomethyl (C) as the fixed modification, oxidation (M) and acetyl (protein N-term) as the variable modification, and MS/MS match tolerance 20 ppm (55). The prediction of the *P. gigenteus* was reanalyzed by combining RNA-Seq analyzed data with Augustus prediction, genemark prediction, and self-trained Augustus model prediction results as our previous study (56). The MaxQuant software was used to calculate the intensity-based absolute quantification (iBAQ) intensities for each protein. Protein identification was supported by at least two unique peptides with a false discovery rate lower than 0.05.

### RNA-Seq

Samples for RNA-Seq analysis were sent to Majorbio Ltd. (Shanghai, China), where the libraries were produced and sequenced. Briefly, total RNA was extracted from *P. giganteus* samples using Trizol reagent (Takara, Japan) according to the manufacturer’s protocols. The RNA concentration was measured using the Nanodrop spectrophotometer (ThermoFisher, United States), and the RNA integrity was determined by Agilent Technologies 2,100 Bioanalyzer (Agilent, CA, United States). mRNA was enriched from total RNA using oligo (dT) coupled to magnetic beads and disrupted to 200–300 bp fragments by ion interruption. The first strand of cDNA fragments was synthesized using RNA fragments as the template with 6-base random primers and reverse transcriptase. The second strand was synthesized using the first strand as the template. Paired-end sequencing libraries and analysis were performed on an Illumina NovaSeq 6,000 platform (Illumina, CA, United States). Library quality was evaluated using the Agilent Technologies 2,100 Bioanalyzer.

### Genome annotation and transcripts identification and data processing

Adaptors were removed from raw data, and then low-quality reads were filtered using fastp software to generate clean reads. To improve the prediction results, the RNA-Seq data and previous prediction results were integrated using EVidenceModeler v r2012-06-25 software (57) as a previous report (56). The former and newly annotated information could be obtained from the Laboratory of Mushroom Prevision Breeding (see text footnote 1). The clean reads were mapped to the *P. giganteus* genome using Hisat2 software (58). The sam files obtained from Hisat2 was sorted and compressed to bam file using samtools software. Map count was calculated with R package Rsubread software. Abundance estimation (TMM) was performed using tools in the Trinity software suite.^3^ Differential expression analyses were analyzed using edgeR software.

### Bioinformatic analysis and data visualization

Proteome analysis was processed using Perseus software v1.6.15.0 (59). Median normalization methods were used to normalize the protein intensities between different groups. The *t*-test method was used to analyze the differential protein expression between the pileus and stem of the *P. giganteus* fruiting body. The normalization method for differential gene expression is weighted trimmed mean of the log expression ratios (trimmed mean of M values (TMM)). The differential expression analysis of gene expression between two groups was performed using run_DE_analysis tools in Trinity software suite with edgeR software (60). Differential expression analysis method between multi groups is a one-way analysis of variance (ANOVA) test, which was performed using stats.f_oneway function from SciPy packages. Correlation analysis, Venn plot, volcano plot, heat map, and PCA analysis were performed and visualized using R packages corrplot, venn, ggplot2, factoextra, and FactoMineR. GO and KEGG enrichment was performed using Fisher’s exact test using ClusterProfiler v 4.4.4 R package (61).

## Data availability statement

The datasets presented in this study can be found in online repositories. The names of the repository/repositories and accession number(s) can be found in the article/Supplementary material.

## Author contributions

HiY, QL and HoY: conceptualization. CS: data curation. NJ and HoY: formal analysis. HiY: funding acquisition. MY and DZ: investigation. MY, XC, and LZ: methodology. CS: supervision. CS and MZ: validation. HoY: writing—original draft. JL and HiY: writing—review and editing. All authors contributed to the article and approved the submitted version.

## Funding

This research was funded by Shanghai Science and Technology Commission Action of Scientific and Technological Creation (23N61900300), China Agriculture Research System (CARS20), SAAS Program for Excellent Research Team ([2022]001), and Shanghai Science and Technology Commission The Belt and Road Project (20310750500).

## Conflict of interest

The authors declare that the research was conducted in the absence of any commercial or financial relationships that could be construed as a potential conflict of interest.

## Publisher’s note

All claims expressed in this article are solely those of the authors and do not necessarily represent those of their affiliated organizations, or those of the publisher, the editors and the reviewers. Any product that may be evaluated in this article, or claim that may be made by its manufacturer, is not guaranteed or endorsed by the publisher.

## Footnotes

^1^ https://file.mushroomlab.cn

^3^ http://trinityrnaseq.github.io
