# Supplementary material for: Comparative analysis of proteomes and transcriptomes revealed the molecular mechanism of development and nutrition of Pleurotus giganteus at different fruiting body development stages
Source: Front Nutr. 2023 Jul 21;10:1197983. doi: 10.3389/fnut.2023.1197983 (PMC10402744; doi:10.3389/fnut.2023.1197983)
Supplement: Supplementary file 1 [file Data_Sheet_1.docx]

**Supplementary Materials**

**Comparative Analysis of Proteomes and Transcriptomes Revealed the Molecular Mechanism of Development and Nutrition of *Pleurotus giganteus* at Different Fruiting Body Development Stages**

Hailong Yu^1,2†^, Ning Jiang^1†^, Miaomiao Yan^1,3†^, Xuan Cheng^4^, Lujun Zhang^1^, DandanZhai^3^, Jianyu Liu^1^, Meiyan Zhang^1^, Chunyan Song^1^, Hao Yu^1,2*^ and Qiaozhen Li^1*^

^1^National Engineering Research Center of Edible Fungi, Institute of Edible Fungi, Shanghai Academy of Agricultural Sciences, Shanghai 201403, China; ^2^Shandong Provincial Key Laboratory of Applied Mycology, School of Life Sciences, Qingdao Agricultural University, 700 Changcheng Road,Chengyang District, Qingdao 266109, Shandong Province, People'sRepublic of China; ^3^Engineering Research Centre of Chinese Ministry of Education for Edible and Medicinal Fungi, Jilin Agricultural University, Changchun, Jilin 130118, China; ^4^Agricultural [Specialty Industry](javascript:;) Development Center, 15 Chayuan Road, Quzhou, Qujiang, 324000, Zhejiang Province, People’s Republic of China.

Running title: *Pleurotus giganteus* fruiting body development

§Hailong Yu, Ning Jiang, and Miaomiao Yan contributed equally to this work.

*Corresponding author:

Qiaozhen Li E-mail: liqiaozhen@saas.sh.cn

Hao Yu E-mail: yuhaosunshine@163.com

**Keywords:** edible mushroom; *Pleurotus giganteus*; transcriptome; development; proteome

**Figure S1**


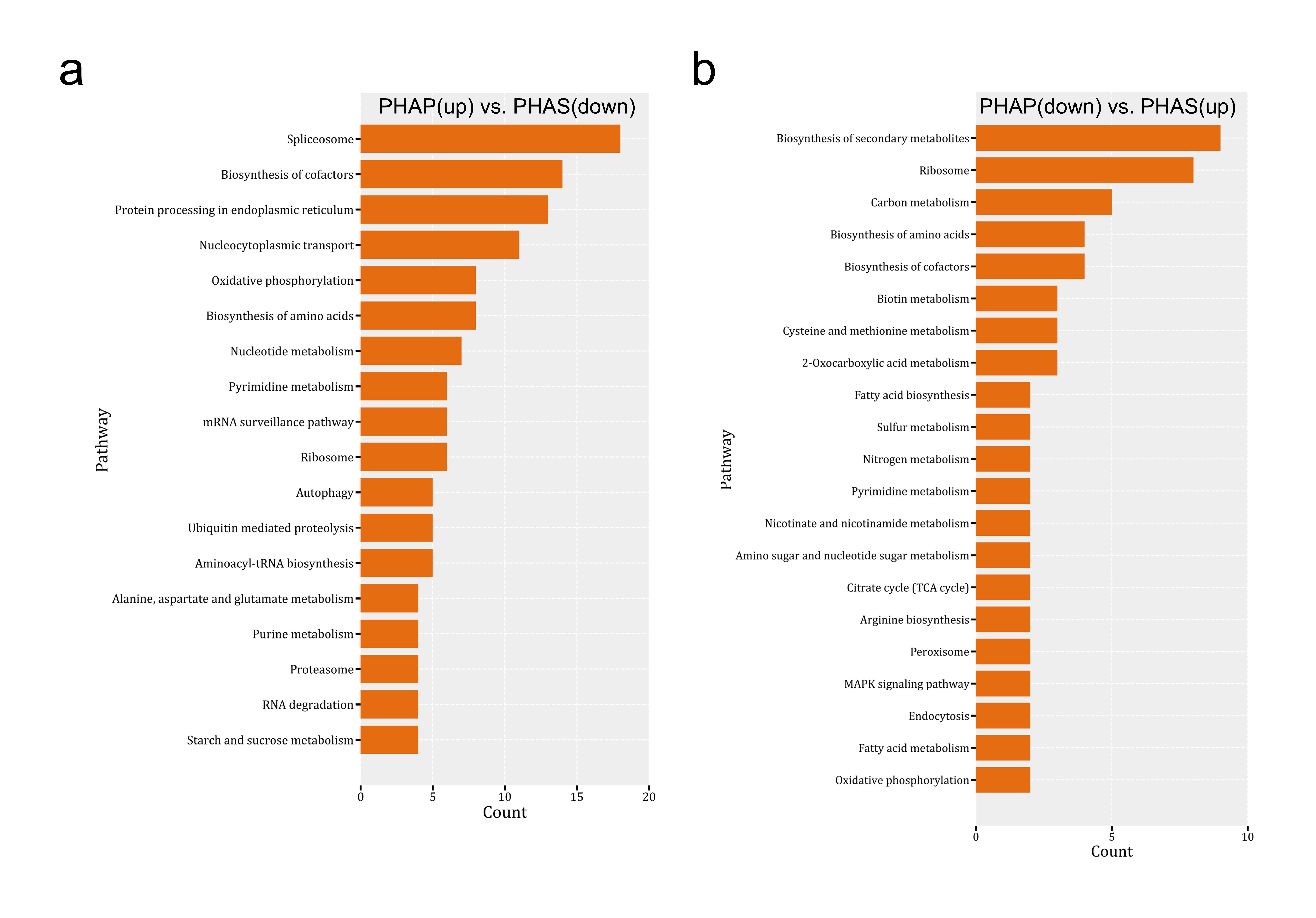


**Enriched KEGG pathways of up-/down-regulated DEPs.** (a) KEGG pathways enriched in up-regulated DEPs in PHAP group. (b) KEGG pathways enriched in down-regulated DEPs in PHAP group.

**Figure S2**


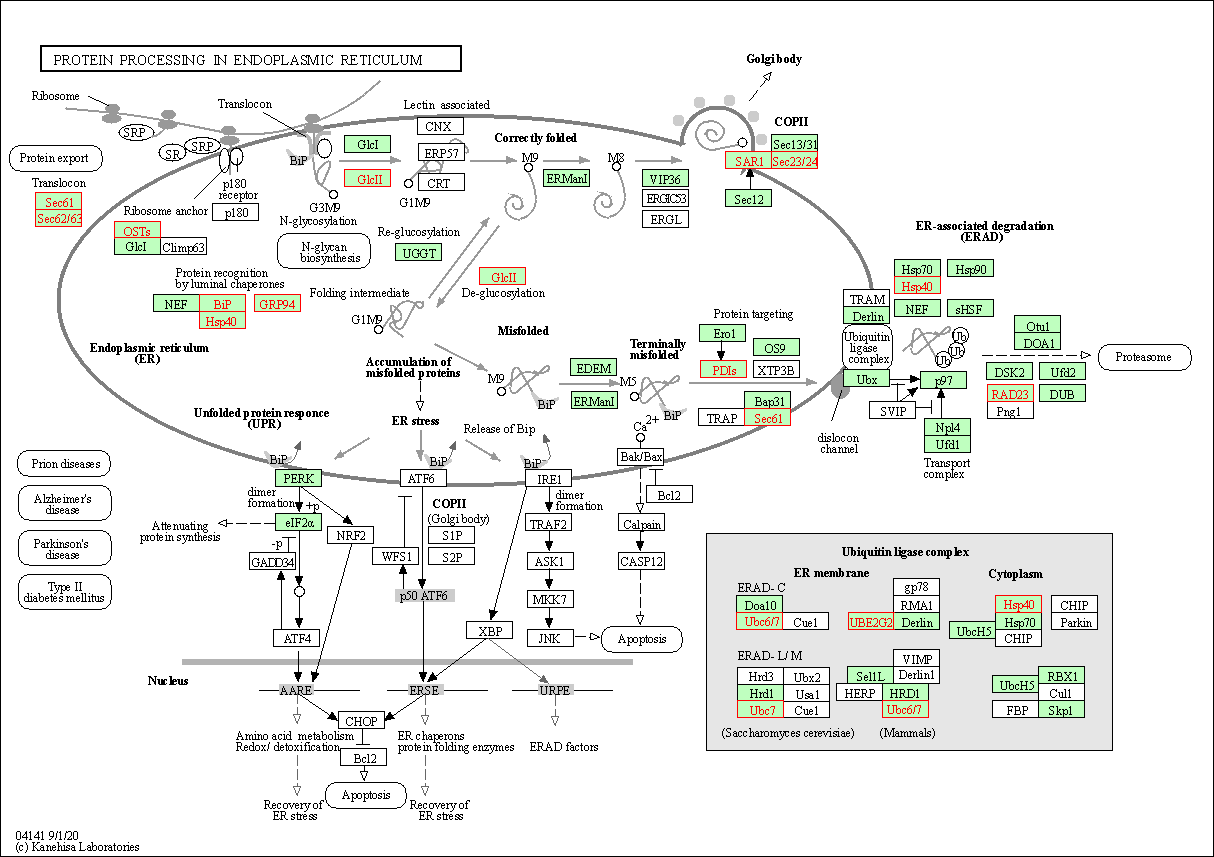


**Representative metabolic pathway map of differentially expressed proteins involved in** **protein processing in endoplasmic reticulum in KEGG pathway.** Red for up-regulated proteins in PHAP group compared with PHAS group.

**Figure S3**


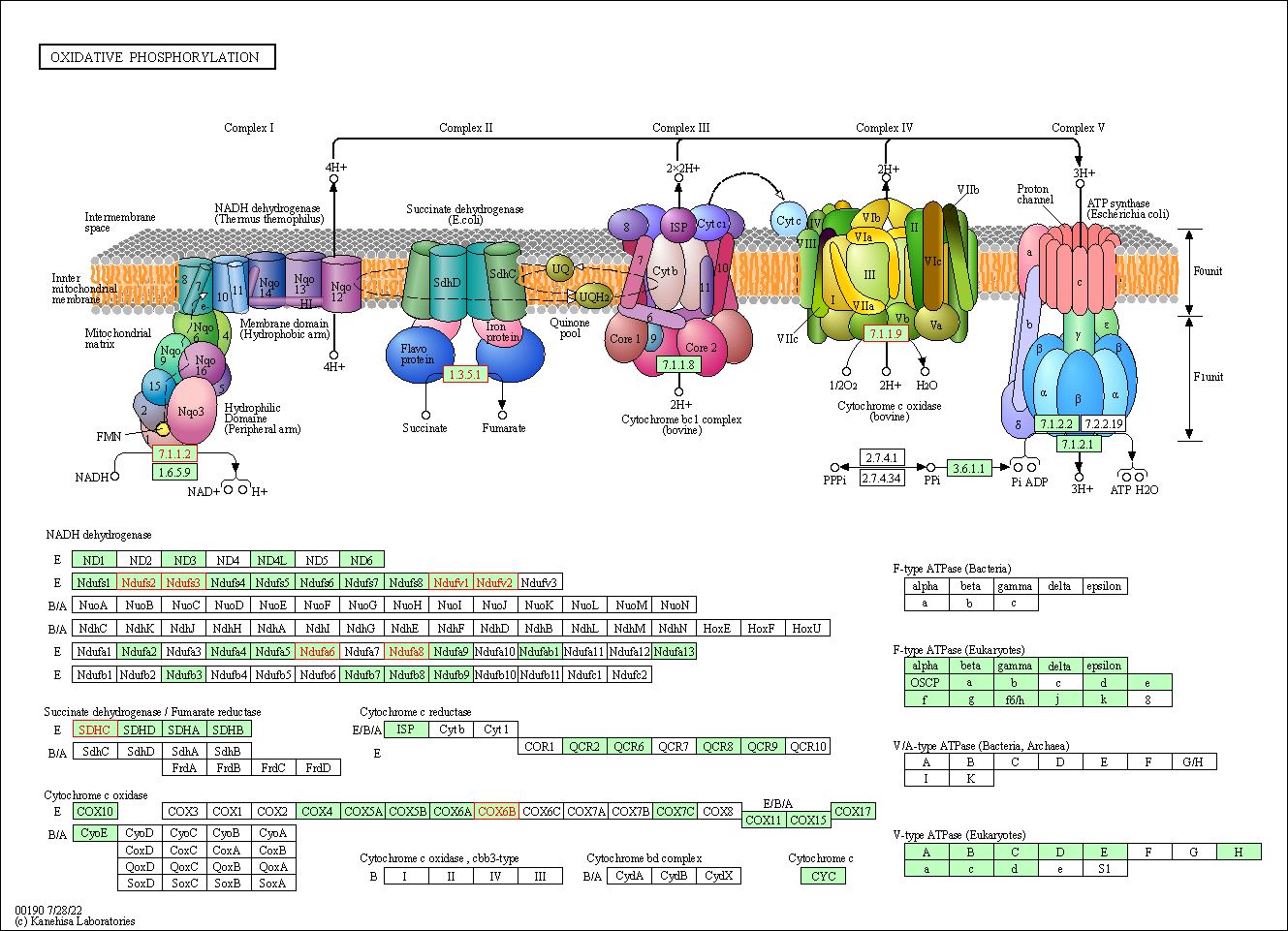


**Representative metabolic pathway map of differentially expressed proteins involved in oxidative phosphorylation in KEGG pathway.** Red for up-regulated proteins in PHAP group compared with PHAS group.
